# The Prognostic Value of Troponin in Pediatric Polytrauma

**DOI:** 10.3389/fped.2019.00477

**Published:** 2019-11-20

**Authors:** Christian Karl Braun, Annika Schaffer, Birte Weber, Markus Huber-Lang, Miriam Kalbitz, Jochen Preßmar

**Affiliations:** ^1^Institute of Clinical and Experimental Trauma-Immunology, University Hospital of Ulm, Ulm, Germany; ^2^Department of Traumatology, Hand-, Plastic-, and Reconstructive Surgery, Center of Surgery, University Hospital of Ulm, Ulm, Germany

**Keywords:** polytrauma, troponin, emergency room, cardiomyopathy, lung contusion, thorax trauma

## Abstract

**Introduction:** Severe trauma accounts for a great number of deaths among children and adolescents. The diagnostic value of troponin serum levels of severely injured patients has been reported for adults, but data on pediatric polytrauma (PT) are scarce. Therefore, we conducted a retrospective monocentered study analyzing the prognostic value of troponin T (TnT) in pediatric trauma patients at the time point of hospital admission.

**Methods:** Data of 88 polytraumatized pediatric patients admitted to the emergency room of the University Hospital of Ulm, Germany, between 2007 and 2016 were analyzed retrospectively. The data source was the written and digital patient records. Interleukin-6 (IL-6), creatine kinase activity (CK activity), and lactate and TnT levels were measured by a certified clinical diagnostic laboratory; and patients were stratified for the Injury Severity Score (ISS). The prognostic value for lung contusion, organ dysfunction, and fatal outcome was statistically explored. The study was approved by the independent ethical committee of the University of Ulm (#44/18).

**Results:** TnT levels were significantly increased in patients after severe PT compared with mild or moderate trauma severity as assessed by ISS values. Patients with TnT levels above the cutoff showed significantly increased levels of IL-6 and CK activity and a significantly prolonged stay in the intensive care unit. However, TnT levels did not correlate with absolute ISS values. TnT levels were significantly increased in patients with chest trauma and lung contusion. The incidence of lung contusion was associated with elevation of TnT. So was the onset of organ dysfunction, defined as a Sequential Organ Failure Assessment (SOFA) score ≥ 2 and fatal outcome, with a significant enhancement of plasma levels in children with organ dysfunction and in non-survivors.

**Conclusion:** These descriptive data suggest that evaluation of TnT on admission of multiply injured children may help in predicting severity of injury and mortality in the clinical course after trauma and thus may be a useful addition to established prognostic parameters in the future.

## Introduction

Severe trauma from road traffic accidents is still one of the leading causes of death in both children and adolescents (1). When thoracic trauma is present among multiple injuries, the overall mortality rises, especially in combination with an additional trauma to the head or abdomen (2). Owing to pending ossification, the ribcage of children is more flexible than that of adults, rendering children more prone to injuries of thoracic organs (3).

In that regard, lung contusion may lead to impairment of oxygenation and subsequently to hypoxemia, often without evident external signs of injury (4). However, although the role of pulmonary contusions in pediatric polytrauma (PT) has been well-characterized, the significance of cardiac impairment is not well-characterized yet, probably owing to its rarity. Nonetheless, blunt cardiac injury in children has been reported to cause significant arrhythmias (5) and even to lead to long-term impairment of cardiac function (6). Moreover, experimental models of trauma have shown notable structural and functional impairment of the heart after traumatic injury (7–9). Furthermore, autoptic studies on adults have revealed marked lesions in heart specimens of polytraumatized adults (10).

In modern clinical practice, cardiomyocyte damage is commonly diagnosed by the highly sensitive and specific cardiac troponins analyzed from patients' plasma, ECG, and echocardiography. Elevated plasma levels of cardiac troponin were associated with the severity of injury and fatal outcome in adult PT patients (11), although its predictive value for diagnosable cardiac injury is widely discussed (12). However, considering pediatric PT, only little is known about the importance of troponin evaluation in the emergency room (ER) and its role in predicting outcome-associated endpoints. In children, a commonly used marker for systemic inflammation is interleukin-6 (IL-6) (13). Recently IL-6 has also been proposed to correlate with injury severity and the onset of organ dysfunction in pediatric patients (14), but in another study, it failed to predict mortality after trauma (15).

Reliable early laboratory parameters of outcome are needed in the clinical management of pediatric PT, especially after blunt trauma mechanisms with the risk of a reduced ratio of evident clinical signs to the physical damage. Here, we present and retrospectively analyzed data from 88 pediatric trauma patients admitted to a level 1 trauma center. The purpose of this study was to evaluate the predictive value of troponin concentrations in serum samples of pediatric patients on admission to the ER and to further elucidate the clinical value of this practical and commonly used biomarker of cardiac damage.

## Materials and Methods

### Data Collection

In this monocentered, retrospective study, data from polytraumatized pediatric patients admitted to the level 1 trauma center of the University of Ulm between the years 2007 and 2016 were analyzed. The data source was the digital and written patient records. A total of 88 cases were included into the study. This study was approved by the Independent Local Ethics Committee of the University of Ulm (#44/18).

### Injury Severity Score, Data Stratification, and Endpoints of the Study

We defined the severity of trauma employing the Injury Severity Score (ISS), first described by Baker et al. (16). To distinguish various degrees of injury severity, all cases were stratified into four groups: ISS group A (ISS-A; included range: 0–15; *n* = 18; no PT by common definition), ISS group B (ISS-B; included range: 16–24; *n* = 37; mild PT), ISS group C (ISS-C; included range: 25–32; *n* = 18; moderate PT), and ISS group D (ISS-D; included range: >32; *n* = 15; severe PT). Those ranges were predefined based on common definitions in polytraumatized adults (ISS ≥ 16 as defined for PT).

In the same manner, cases were stratified into groups of age: infants (range: <12 months; *n* = 4; median age: not determined), toddlers (range: 1–5 years; *n* = 25; median age: 3), young children (range: 6–12 years; *n* = 41; median age: 8), and adolescents (range: 13–18; *n* = 18; median age: 14) for descriptive epidemiology. The diagnosis of thorax trauma was confirmed by the combined evaluation of the clinician, trauma imaging, and medical history. Lung contusion was radiologically confirmed by ultrasound examination and/or computed tomography. For estimating the onset of organ dysfunction, the Sequential Organ Failure Assessment (SOFA) score was employed as laid out in recent consensus definitions (17). Organ dysfunction was defined as a SOFA score of ≥2.

### Analytical Parameters

IL-6 and cardiac specific troponin T (TnT) were determined by the clinical routine diagnostic laboratory by electro-chemiluminescence immunoassay (ECLIA, Roche). TnT levels were considered “elevated” at values above the cutoff of 14 ng/ml. This cutoff was preset from the clinical routine diagnostic laboratory of the University Hospital of Ulm for adults and was employed in the lack of reliable cutoff values for children. Activity levels of creatine kinase were analyzed by spectrophotometry in the clinical routine diagnostic laboratory. Lactate levels were obtained by point-of-care blood gas analysis.

### Statistical Analysis

Descriptive statistics and statistical testing were performed with SPSS Statistics (Version 24, IBM Corp.). Descriptive statistics and distribution analysis were performed before statistical testing. Gaussian distribution could not be assumed; therefore, only nonparametric tests were used. There was no testing for outliers.

For statistical comparison and analysis of two groups, the Mann–Whitney *U* test was applied. For statistical comparison and analysis of more than two groups, a one-way analysis of variance (ANOVA) on ranks (Kruskal–Wallis analysis) with Bonferroni adjusted *post-hoc* testing for multiple comparisons was performed. Correlation analysis was executed with Spearman's correlation test. Receiver operating characteristic (ROC) analysis was performed to estimate the area under the curve (AUC) and to evaluate sensitivity and specificity of different cutoff values of TnT and IL-6. Results were considered statistically significant at *p*-values < 0.05.

For graphical depiction, GraphPad Prism (Version 8, GraphPad Software, Inc.) was used. Epidemiological data are presented as grouped absolute counts of cases, and statistical data are presented as median and quartiles (obtained by weighted average algorithm with interpolation; values were rounded if necessary).

## Results

### Descriptive Epidemiological Data

In total, 88 cases of pediatric PT admitted to the University Hospital of Ulm were included in the present retrospective analysis. The median age was 7.5 years (quartiles: 4; 12) with median ISS of 18.5 (quartiles: 16; 28.5). Of those children, 30 were female and 58 were male.

**Figure 1:**
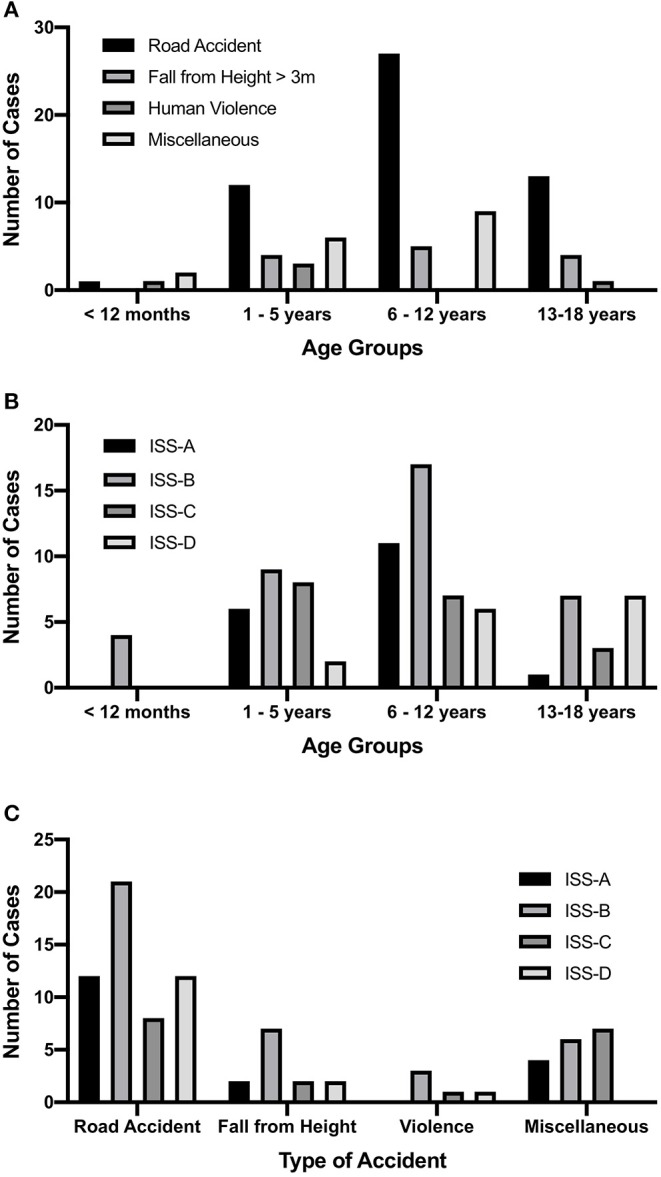
Descriptive epidemiological data of the included cases. **(A)** Number of cases for each type of accident, sorted by age groups. The most frequent type of accident in all age groups but infants were road traffic-associated accidents. Falls from height < 3 m, sporting accidents, animal attacks, and so were put together as “miscellaneous.” **(B)** Number of cases for each Injury Severity Score (ISS) group sorted by age. In all groups, there was an approximately parametric distribution of cases. At higher age, a weak association with higher ISS values was observable. **(C)** Number of cases for each ISS group sorted by a type of accidents. No distinct pattern as to which type was associated with more severe trauma could be detected. ISS-A, ISS group A (included range: 0–16; *n* = 18); ISS-B, ISS group B (included range: 16–24; *n* = 37); ISS-C, ISS group C (included range: 25–32; *n* = 18); ISS-D, ISS group D (included range: >32; *n* = 15).

Among the distinct types of injury, road accidents were most common (*n* = 53), followed by falls from height > 3 m (*n* = 17). Another five cases were caused by human violence. Rarer types of injuries, such as by animal attack, fall from heights < 3 m, and sporting accidents, were put together as “miscellaneous” (*n* = 17). Road accidents were also the most common type of injury in all age groups except in infants (Figure 1A).

In each age group, the majority of cases were classified as ISS-B, with a slight tendency of more severe ISS values with increasing age (Figure 1B). However, there was no statistically significant difference between the medians of age in the ISS groups (*p* = 0.059, data not shown), and vice versa the Kruskal–Wallis analysis did fail to show a significant difference in ISS values among age groups (*p* = 0.186, data not shown). Also, no distinct pattern as to which type of injury was associated with the highest values of ISS (Figure 1C) was apparent. As observed for age groups, no significant difference in ISS values among the types of injury has been detected (Kruskal–Wallis analysis; *p* = 0.861, data not shown).

### Children With Severe Polytrauma Showed Markedly Enhanced Levels of Troponin T, Interleukin-6, and Creatine Kinase Activity and Higher Incidences of Lung Contusions, Organ Dysfunction, and Fatal Outcome

Stratified ISS groups were analyzed for differences in analytical parameters. Plasma levels of IL-6 (*n* = 35), an early pro-inflammatory cytokine commonly used for the measurement of early systemic inflammation in pediatric patients, showed a significant difference between ISS-D and ISS-C (*p* = 0.022; Kruskal–Wallis analysis and Bonferroni *post-hoc* testing for multiple comparisons), but no difference among the other ISS groups (Table 1). A very similar pattern was observed for plasma levels of TnT (*n* = 34), a specific marker of cardiac damage (Table 1), with significantly enhanced plasma levels in the ISS-D group compared with ISS-C (*p* = 0.002; Kruskal–Wallis analysis and Bonferroni *post-hoc* testing for multiple comparisons). As an unspecific marker of muscle cell damage, creatine kinase (CK) activity (*n* = 50) was likewise increased in the ISS-D group compared with both ISS-C (*p* = 0.001) and ISS-B (*p* = 0.026; both Kruskal–Wallis analysis and Bonferroni *post-hoc* testing for multiple comparisons). Lactate levels (*n* = 37), obtained by blood gas analysis in the ER, were enhanced in patients after moderate (ISS-C; *p* = 0.032 vs. ISS-B) and severe (ISS-D; *p* = 0.02 vs. ISS-B; both Kruskal–Wallis analysis and Bonferroni *post-hoc* testing for multiple comparisons) PT (Table 1).

**Table 1:** Stratification of polytraumatized patients according to trauma severity and analysis of differences in analytical and outcome parameters among groups.

		**Groups**				
		**ISS-A**	**ISS-B**	**ISS-C**	**ISS-D**	
	Range	<16	16–24	25–32	>32	
	*n*	18	37	18	15	
	Median ISS	10	17	26	38	
	Median age	7.5	6	6.5	12	
**Parameter**	***n*** **(included cases)**^b^					**Kruskal–Wallis analysis of variances**^c^
**IL-6 (median [1./3. Qrt]; pg/ml)**	35	63 (10/150)	54 (24/104)	5 (2/76)	267 (82/709)	*p* = 0.032
**TnT (median [1./3. Qrt]; ng/ml)**	34	5 (4/10)	8 (0/20)	0 (0/4)	84 (15/837)	*p* = 0.005
**CK (median [1./3. Qrt]; U/L)**	50	362 (199/1115)	381 (211/590)	191 (100/382)	1518 (737/2497)	*p* = 0.002
**Lactate (median [1./3. Qrt]; mmol/L)**	37	1.6 (1.1/1.9)	1.1 (0.7/1.9)	3.1 (1.7/3.25)	2.5 (1.8/5.4)	*p* = 0.005
**Length of ICU (median [1./3. Qrt]; days)**	85	1.5 (1/3)	3 (2/5)	3.5 (2/7)	5 (3/9)	*p* = 0.004
**Thorax trauma/cases (fraction)**	87	8/18 (0.44)	20/36 (0.56)	8/18 (0.44)	14/15 (0.93)	^§^
**Lung contusion^a^/cases (fraction)**	64	4/15 (0.27)	14/28 (0.5)	3/10 (0.3)	9/11 (0.81)	^§^
**SOFA ≥ 2/Cases (fraction)**	77	2/15 (0.13)	12/35 (0.34)	4/15 (0.27)	11/12 (0.92)	^§^
**Fatal outcome/cases (fraction)**	88	0/18 (0.0)	0/37 (0.0)	1/18 (0.06)	5/15 (0.33)	^§^

§ *, significant association via chi-square analysis; ISS, Injury Severity Score; IL-6, interleukin-6; TnT, troponin T; CK, creatine kinase; SOFA, Sequential Organ Failure Assessment*.

a *Radiologically confirmed*.

b *Depicts the number of cases for which data were available*.

c *Column depicts overall significances*.

More severe PT was associated with longer median periods in the intensive care unit (ICU; *n* = 85; Table 1). Higher ISS values and categorization into the ISS-D group were significantly associated with greater likelihood of thorax trauma and lung contusion as confirmed by radiological diagnostics (chi-square testing for association; *p* < 0.05; Table 1). The same significant association could be obtained for increased SOFA-scores (≥2) and fatal outcome (chi-square testing for association; *p* < 0.05; Table 1).

### Elevated Troponin T Is Associated With Higher Levels of Interleukin-6 and Creatine Kinase Activity and Prolonged Stay in the Intensive Care Unit

To test whether patients with TnT values above the cutoff also show differences in other parameters of interest, we divided cases into a group with TnT values below the cutoff (TnT–; median/interquartile range: 4/7 ng/ml; *n* = 22) and with increased values above the cutoff (TnT+; median/interquartile range: 41/103 ng/ml; *n* = 12). Group medians were tested for statistical significance, using the Mann–Whitney *U* test for nonparametric testing.

**Figure 2:**
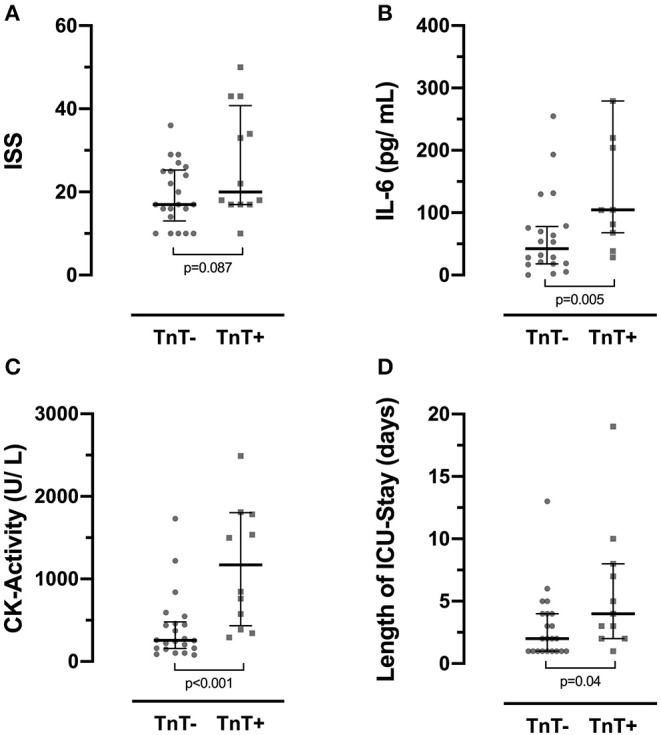
Cases were stratified into two groups, those with plasma TnT levels below (TnT–) and above (TnT+) a set cutoff of 14 ng/ml. **(A)** Injury Severity Score (ISS) values did not significantly differ between groups. **(B)** Interleukin 6 (IL-6) and **(C)** creatine kinase activity (CK activity) levels were significantly increased in the TnT+ group. **(D)** Further, the TnT+ group presented with a significant increase of the median length of stay in the intensive care unit (ICU). Data depict median and quartiles. In **(B)**, two data points (IL-6; subject 52: >5,000 pg/ml; subject 61: 709 pg/ml) in the TnT+ group are not shown for better visualization. In **(C)**, one data point (CK activity; subject 52: 5,881 U/L) in the TnT+ group is not shown for better visualization. TnT, troponin T.

There was no significant correlation between TnT plasma levels and ISS values (Spearman's correlation; *r* = 0.278; Table 2), and patients with TnT levels above the cutoff failed to show significantly increased ISS values (Figure 2A). Notably, both IL-6 (*n* = 31) and CK activity (*n* = 34) levels were significantly increased in TnT+ group (Figures 2B,C) and correlated with measured TnT levels in plasma (Spearman's correlation; TnT vs. IL-6: *r* = 0.558; TnT vs. CK activity: *r* = 0.748; Table 2). In contrast, group means of lactate levels on admission did not show significant differences between the TnT– and TnT+ groups and failed to display any correlation (Table 2; no graphical depiction). The length of ICU stay was significantly longer in the TnT+ group, but only a weak correlation between days of ICU treatment and TnT levels was observable (Figure 2D; Table 2).

**Table 2:** Correlation analyses of troponin T (TNT) levels in patients' plasma with plasma levels of interleukin-6 (IL-6), creatine kinase activity levels (CK activity), and lactate levels; Injury Severity Score (ISS) values; and length of stay in the intensive care unit (ICU).

	***n* (included cases)^a^**	**Spearman's *r***	***p*-value**
**ISS**	34	0.278	n.s.
**IL-6**	31	0.558	0.001
**CK activity**	34	0.748	<0.001
**Lactate**	12^b^	−0.200	n.s.
**Length of ICU**	34	0.320	n.s.

*n.s., not significant*.

a *Depicts the number of cases for which data were available*.

b *Owing to low case numbers, data should be interpreted with caution*.

On categorizing cases into patients with IL-6 values either below (*n* = 6; median/interquartile range: 2/4 pg/ml) or above (*n* = 29; median/interquartile range: 76/132 pg/ml) a set cutoff of 7 pg/ml, statistical comparison of means/medians of the above-mentioned parameters did not show any significance. IL-6 values did correlate with CK activity (Spearman's correlation; *r* = 0.728), but not with the other parameters of interest (Supplementary Table 1).

### Troponin T Values Are Significantly Increased in Children With Thorax Trauma, Lung Contusions, Organ Dysfunction, and Fatal Outcome

**Figure 3:**
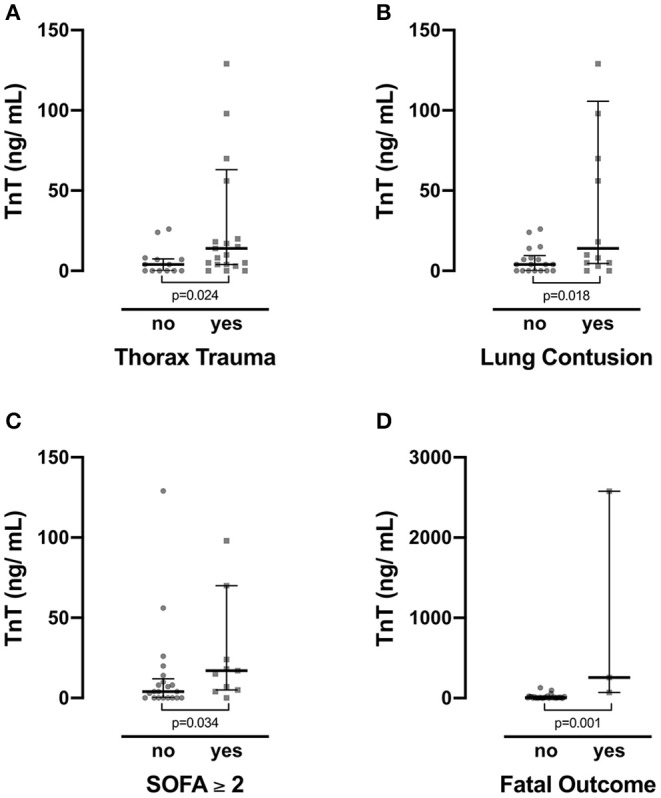
Troponin T (TnT) levels were compared for various cohorts. Patients with **(A)** thorax trauma and **(B)** lung contusions showed significantly increased levels of TnT. **(C)** Also, in the cohort with organ dysfunction as defined by a Sequential Organ Failure Assessment (SOFA) score ≥ 2, TnT levels were enhanced. **(D)** Finally, non-survivors presented with significantly increased TnT levels than did survivors. Data depict median and quartiles. In **(A,B)** two data points (TnT; subject 52: 2,577 ng/ml; subject 61: 257 ng/ml) in the groups depicting the presence of thorax trauma and lung contusion are not shown for better visualization. In **(C)** one data point (TnT; subject 61: 257 ng/ml) in the group with a SOFA score ≥ 2 is not shown for better visualization.

Among all cases, 50 patients had a reported thorax trauma (37 patients had no reported thorax trauma; in one case, the information was missing). As expected, among patients with thorax trauma, a higher fraction was above the set cutoff for TnT levels (10/*n* = 21, fraction: 0.47) than among patients without reported thorax trauma (2/*n* = 11, fraction 0.18), although the association did fail to show a significant value on chi-square testing (*p* = 0.056; Supplementary Table 2). Mean TnT level of patients with thorax trauma was significantly higher than in the group without reported thorax trauma (Figure 3A). On ROC analysis, the AUC for TnT/thorax trauma was 0.73 (Supplementary Figure 1A).

Of all cases, 30 patients had a reported lung contusion diagnosed by a radiologist. A fraction of 0.5 (7/*n* = 14) of the patients with radiological detectable lung contusion had elevated TnT levels, whereas only a fraction of 0.17 (3/*n* = 18) of all cases without contusions showed TnT levels above the set cutoff (Pearson's chi-square: *p* = 0.044; Supplementary Table 2). Patients with reported lung contusions had a significantly higher TnT levels than patients without reported lung contusion (Mann–Whitney *U* test for nonparametric testing; *p* = 0.18; *n* = 32; Figure 3B). On ROC analysis, the AUC for TnT/lung contusion was 0.74 (Supplementary Figure 1B).

Similarly, patients with a SOFA score ≥ 2 on admission had significantly enhanced TnT levels than had patients with a SOFA score of <2 (Mann–Whitney *U* test for nonparametric testing; *p* = 0.034; *n* = 32; Figure 3C). A fraction of 0.64 (7/*n* = 11) of the patients with a SOFA score ≥ 2 had TnT levels above 14 ng/ml, whereas only 0.19 (4/*n* = 21) of the patients without SOFA score values of <2 had elevated TnT levels. Upon testing Pearson's chi-square, this association was significant (*p* = 0.012). On ROC analysis, the AUC for TnT/organ dysfunction was 0.73 (Supplementary Figure 1C).

Of all cases, six patients died after admission (*n* = 5 after road accidents; *n* = 1 after a fall from height > 3 m). Patients with fatal outcome showed significantly increased TnT levels than did survivors (Mann–Whitney *U* test for nonparametric testing; *p* = 0.001; *n* = 34; Figure 3D). TnT elevation above the set cutoff was significantly associated with fatal outcome (Pearson's chi-square: *p* = 0.014; Supplementary Table 2). Moreover, none of the non-surviving patients presented TnT levels below the cutoff (calculated negative predictive value: 1.0). On ROC analysis, the AUC for fatal outcome was 0.98 (Supplementary Figure 1D).

In comparison, IL-6 did not show a significant correlation for the prevalence of thorax trauma and/or lung contusion nor for organ dysfunction. Fractions of patients in the respective groups were highly similar for these endpoints (Table 3). However, the fraction of patients with IL-6 values above the cutoff showed a significant correlation with the endpoint of fatal outcome (Table 3). On ROC analysis, the AUC for IL-6/fatal outcome was 0.90 (Supplementary Figure 2).

**Table 3:** Prognostic value and association analysis for IL-6 plasma levels elevated above the cutoff of 7 pg/ml.

	**Thorax trauma**		
	**Not reported**	**Reported**	
***n*** **(included cases)^c^**	14	21	
**IL-6 (median [1./3. Qrt];** **pg/ml)**	54 (13/105)	68 (23/131)	n.s.^a^
**Above cutoff/cases (fraction)**	11/14 (0.78)	18/21 (0.85)	n.s.^b^
	**Lung contusion**		
	**Not reported**	**Reported**	
***n*** **(included cases)**^**c**^	19	21	
**IL-6 (median [1./3. Qrt];** **pg/ml)**	53 (17/106)	72 (21/118)	n.s.^a^
**Above cutoff/cases (fraction)**	15/19 (0.79)	12/14 (0.86)	n.s.^b^
	**SOFA score**		
	**0–2**	**>2**	
***n*** **(included cases)**^**c**^	22	11	
**IL-6 (median [1./3. Qrt];** **pg/ml)**	54 (17/93)	60 (17/261)	n.s.^a^
**Above cutoff/cases (fraction)**	18/22 (0.82)	9/11 (0.82)	n.s.^b^
	**Fatal outcome**		
	**Survivor**	**Non-survivor**	
***n*** **(included cases)**^**c**^	32	3	
**IL-6 (median [1./3. Qrt];** **pg/ml)**	53 (17/105)	395 (82/709)	*p* = 0.020^a^
**Above cutoff/cases (fraction)**	26/32 (0.81)	3/3 (1.0)	n.s.^b^

*n.s., not significant; IL-6, interleukin-6; SOFA, Sequential Organ Failure Assessment*.

a *Statistical analysis with Mann–Whitney U test for nonparametric testing*.

b *Statistical association analysis with chi-square test*.

c *Depicts the number of cases for which data were available*.

## Discussion

In this study, we present retrospectively analyzed data from a total of 88 cases of pediatric PT admitted to our level 1 trauma center. Epidemiological exploration revealed road traffic-associated accidents as the most frequent type of accident in our study population followed by falls from large height, similar to findings from previous publications on pediatric PT (3, 6, 18). A significant association of the incidence of thorax trauma and lung contusions with the stratified ISS was found. Additionally, fractions of patients with organ dysfunction as determined by a SOFA score ≥ 2 and fatal outcome were significantly higher in the ISS-D group than in other groups.

So far, there are only few studies on the performance of the ISS as a scoring tool for severely injured children. Although evaluating the ISS in pediatric PT was not within the primary scope of this study, our data suggest that the ISS performs less reliably in discriminating between less severe forms of trauma in children. This is in accordance with a large study, showing that higher cutoff values of the ISS are needed to define severe trauma in children (19).

As expected, we found significantly increased levels of IL-6 and CK activity in the group with ISS values above 32. IL-6 is a proinflammatory cytokine, which, besides IL-8, is commonly used in pediatric clinical practice to monitor systemic inflammation and bacterial infection (13). Recently, initial IL-6 has been proposed to correlate with injury severity and onset of multiorgan dysfunction syndrome (MODS) in children (14). In the present patient collective, IL-6 did correlate with CK activity but failed to do so with absolute ISS score values. Interestingly, mean IL-6 values did not differ between groups of mild and moderate trauma (ISS-B vs. ISS-C) but showed a significant enhancement in the group categorized as severe trauma (ISS-D). Further, in this study, IL-6 values were not reliable in predicting an increased SOFA score as an indicator of multiorgan dysfunction. Published data on the predictive value of IL-6 on organ dysfunction after pediatric PT are scarce, and a recent report only found consistent correlations of IL-6 with MODS on days 2 and 3 after admission (14), suggesting a more important role of this parameter in the consecutive course after trauma. We found significantly increased levels of IL-6 in non-survivors compared with survivors. However, elevation of plasma levels above the cutoff of 7 pg/ml did not correlate well with fatality, and ROC analysis showed very low specificity (~0.16, data not shown) for this cutoff value. These data match previous findings, where IL-6 was demonstrated not to be a good prognostic marker for mortality after pediatric trauma (15). Whether IL-8, another early marker of systemic inflammation, would be of greater value could not be obtained from our data source and has to be determined in future studies. Whereas in adults, IL-6 plasma levels have been described to correlate with severity of injury (20) and also with the severity of chest trauma (11), the significance of IL-6 levels in traumatized children remains to be defined.

Creatine kinase activity is a nonspecific marker of overall muscle damage. We observed markedly enhanced CK activity levels in the ISS-D groups but no significant difference between groups with mild and moderate injury as obtained by ISS values. To the best of our knowledge, there is no published literature analyzing the predictive value of CK analysis in pediatric PT so far. Very high CK levels were reported to be associated with acute renal failure and persistent renal insufficiency after traumatic injuries and mechanical muscle damage in adults (21).

As for CK activity, literature is only scarcely available for the diagnostic value of lactate by point-of-care analysis in polytraumatized children. We observed significantly enhanced lactate concentrations after moderate (ISS-C) and severe (ISS-D) compared with mild PT but no distinction between those groups. Hwabejire et al. identified lactate levels obtained in the ER as predictive for fatal outcome in the age group of younger adults (16–44 years of age) (22), but as by the age of the patient collective, comparability to our data is questionable. More research is needed to clarify the importance of measuring lactate levels of children in the ER.

As a main focus in this study, we observed significantly increased plasma levels of TnT in ISS-D group as compared with the ISS-A, ISS-B, and ISS-C groups. Interestingly, mean TnT levels did not differ between patients with mild (ISS-B) and moderate (ISS-C) trauma, suggesting a diagnostic role of TnT especially for patients with severe trauma. This is in accordance to the weak correlation of TnT levels with ISS values in this study. It is noteworthy, that, whereas distinguishing the patients for whom data on TnT levels were available by a clinically used cutoff (14 ng/ml) showed significant differences between those groups regarding IL-6 levels, CK activity, and length of ICU stay (Figure 2), those parameters showed only a weak correlation with individual TnT levels. This suggests, that trauma severity alone is not sufficient to account for higher TnT levels, and it is tempting to speculate that other yet unknown factors drive TnT release in the early phase after trauma. Experimental studies may elaborate the mechanical background behind this observation.

In the present cohort, children with elevated TnT levels (as defined by levels above a cutoff of 14 pg/ml) showed significantly increased IL-6 and CK activity levels with positive correlations among these parameters. This suggests an overall association of increased TnT levels with more enhanced tissue damage and systemic inflammation. Pathophysiologically, it is not possible at this point to determine whether more severely injured children simply have a higher risk for concomitant direct cardiac injury or if a status of enhanced systemic inflammation further damages cardiac cells via humoral mediators or both. In sepsis, cardiac impairment through systemic inflammation is well-described in adults [see (23, 24) for corresponding reviews]. In experimental studies, the role of various mediators, such as ILs (25, 26), reactive oxygen species (27), complement components (28), and damage-associated molecular patterns (DAMPs) (29, 30), were described to impair the function of the myocardium. Regarding the increased CK activity levels, it is tempting to speculate that with increased muscle cell loss, more DAMPs are released into the bloodstream, causing remote organ damage in general and impairment of the cardiac muscle cells in particular. In experimental *in vivo* trauma models, functional impairment, and morphological changes of the heart could be observed as well (7–9).

To what extent the enhanced TnT levels in the present study reflect cardiac damage with functional consequences could not be drawn from our data, as only two children had echocardiographic evaluation on admission.

To investigate the prognostic value of TnT in severely injured pediatric patients, we analyzed three endpoints in this study: (1) the incidence of lung contusions (as diagnosed by radiological imaging), (2) the onset of organ dysfunction as obtained by a SOFA score of ≥2, and (3) fatal outcome in the course of treatment.

In our study cohort, 50 patients suffered a reported chest trauma. In this group, TnT levels were significantly increased. However, there was only a weak association between TnT elevation above the cutoff and the incidence of thorax trauma, and less than half of the patients showed increased TnT levels. These findings suggest that injury of the thorax alone does not fully account for TnT elevation in plasma but is associated with higher absolute values. Notably, positive TnT plasma levels were significantly associated with the incidence of lung contusions in the present study. Pulmonary injury in severely injured children is common and aggravates the clinical outcome by ventilation impairment and by rendering the lung prone to subsequent infection and respiratory distress syndrome (4, 31). The value of cardiac specific troponins for the diagnosis of lung contusions in children has been proposed before (32). Also, lung sonography has been proposed as a diagnostic tool with certain limitations in adults (33) and has previously been described to be useful in the detection of pediatric lung contusion after trauma (34). The combination of sonography with ER evaluation of TnT plasma levels may present a valuable and easy-to-assess duo in diagnosing pediatric lung contusion in the ER. Future research would have to address the question of whether troponin levels alone or in combination with sonography would have sufficient sensitivity and specificity, to spare children of unnecessary radiation exposure from computer tomographic examination, which is the current standard in the diagnosis of lung contusion.

As opposed to lung contusion, cardiac contusion was repeatedly reported to be rare in children (6, 35, 36), so the specificity of troponins for direct cardiac damage after trauma may be questioned. In a large postmortem study, only 41 of 282 children showed signs of cardiac injury at autopsy, and most of them deceased at the scene or on admission (35). These numbers are contradictive with the high fraction of cases with elevated TnT in our study. One possible explanation would be cellular or subcellular impairment of cardiac cells beyond the detectability of common clinical imaging and pathohistological examination.

In a rodent model of PT, troponin elevation was reported in the absence of morphological signs of damage (7). The discrepancy between observed elevations of troponins and tangible cardiac impairment may at least in part be explained by the theory that cardiac cells contain mobilizable cytosolic pools of troponin (37). However, the pathophysiological triggers and mechanisms leading to the release of cardiac troponins are complex and as of yet not completely understood (38). Moreover, it would be of interest whether the hemodynamic status of the patients influences early TnT release. Hemorrhagic shock is known to aggravate organ dysfunction in adults (39). As only a minor fraction of our patients presented with manifest shock on admission (data not shown), we were not able to address this question in this study.

In adults, blunt cardiac injury can lead to arrhythmias (40), with elevated troponin levels being predictive for their incidence (41). Such arrhythmias have been described before (5). Experimental studies on trauma have revealed disintegration of the cardiac gap junctions and dislocation of the important coupling protein Cx43 (7, 8), findings similar to those in septic patients (42). Theoretically, this mechanism may also be involved in a post-traumatic impairment of the electric conduction system of the heart and consecutive arrhythmias.

To what extent the experimental findings translate into clinical consequences, especially in pediatric patients, is not well-characterized. In a recent study on patients admitted to a pediatric ICU, troponin levels proved to be a valuable predictor of mortality when analyzed on admission (43). The same prognostic value for troponins was also reported for adult trauma patients (11, 44). In our study, TnT plasma levels on ER admission were significantly increased in non-survivors, and TnT elevation above the cutoff was significantly associated with fatal outcome. However, these findings need to be interpreted with caution, as TnT levels were only available for three fatal cases. For all endpoints of the study, the data suggest that additional diagnostic evaluation of TnT plasma levels performs better than sole evaluation of IL-6 levels in regard to prognostic statements. Also, TnT may prove as a valuable new marker for lung contusions in severely injured children.

A notable limitation of this study is the lack of functional data to objectify the functional consequences of these results. However, based on our findings, it seems crucial that future research should address the functional implications of the presented observations by means of echocardiography and electrocardiography in a prospective controlled manner. Also, it is not yet clear whether the involvement of other body regions (e.g., the central nervous system and the abdomen) and their respective injury patterns contribute in a specific manner to the release of TnT early after trauma.

## Conclusion

In this study, we present retrospective data on the role of troponin evaluation in polytraumatized children on admission to the ER. Troponin values were significantly increased in patients with severe PT, defined as an ISS of >32 (ISS-D group). TnT plasma concentrations were also associated with higher levels of IL-6 and CK activity in plasma and with prolonged stay in the ICU. Furthermore, children with radiologically confirmed lung contusion and organ dysfunction and non-survivors showed significantly increased levels of TnT. In this study, TnT level evaluation from patients' plasma was more reliable than the analysis of IL-6 levels. Additionally, given the specificity of cardiac troponins for cardiac cell damage, these findings suggest a first descriptive evidence for direct or remote involvement of the heart in pediatric PT and a prognostic value of TnT for the outcome of children with multiple injuries. Nonetheless, the exact pathophysiological mechanisms involved and the functional implications of the reported data are still uncertain and will need careful further evaluation in future prospective studies.

## Data Availability Statement

All original data will be made available by the corresponding author upon reasonable request from qualified researchers.

## Ethics Statement

The studies involving human participants were reviewed and approved by Independent Ethical Committee of the University of Ulm. Written informed consent to participate in this study was provided by the participants' legal guardian/next of kin.

## Author Contributions

CB designed the study, analyzed the data, performed statistical testing, and prepared the manuscript. AS collected and organized the data and critically reviewed the manuscript. BW designed the study and critically reviewed the manuscript. MH-L provided advice for the study design and statistical testing and critically reviewed the manuscript. MK designed the study, critically reviewed the manuscript, and supervised the study. JP designed the study, analyzed the data, prepared the manuscript, and supervised the study.

### Conflict of Interest

The authors declare that the research was conducted in the absence of any commercial or financial relationships that could be construed as a potential conflict of interest.
