# Supplementary material for: The Prognostic Value of Troponin in Pediatric Polytrauma
Source: Front Pediatr. 2019 Nov 20;7:477. doi: 10.3389/fped.2019.00477 (PMC6879657; doi:10.3389/fped.2019.00477)
Supplement: Supplementary file 1 [file Presentation_1.PPTX]

## Slide 1
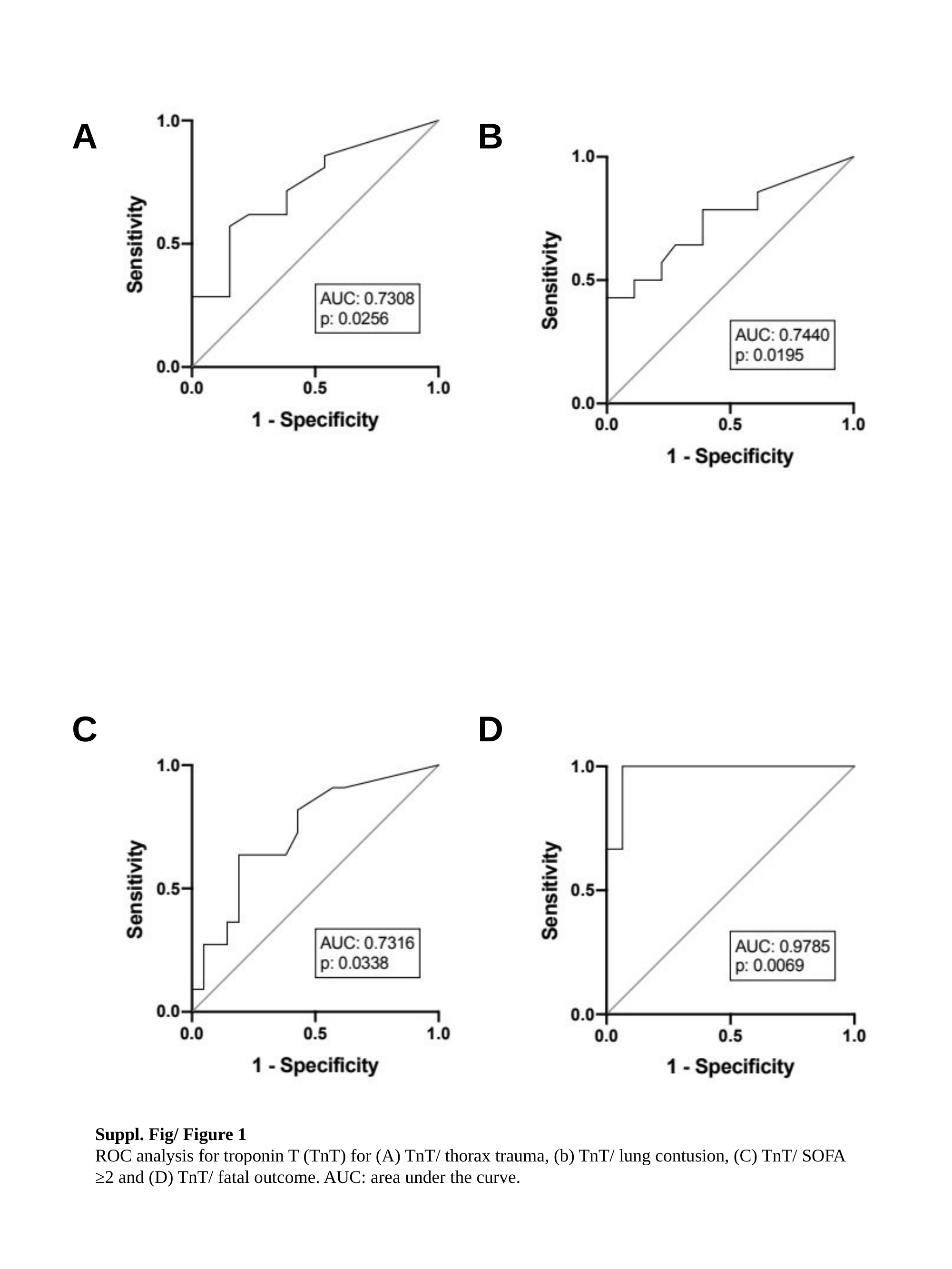

B
A
D
C
Suppl. Fig/ Figure 1
ROC analysis for troponin T (TnT) for (A) TnT/ thorax trauma, (b) TnT/ lung contusion, (C) TnT/ SOFA ≥2 and (D) TnT/ fatal outcome. AUC: area under the curve.

## Slide 2
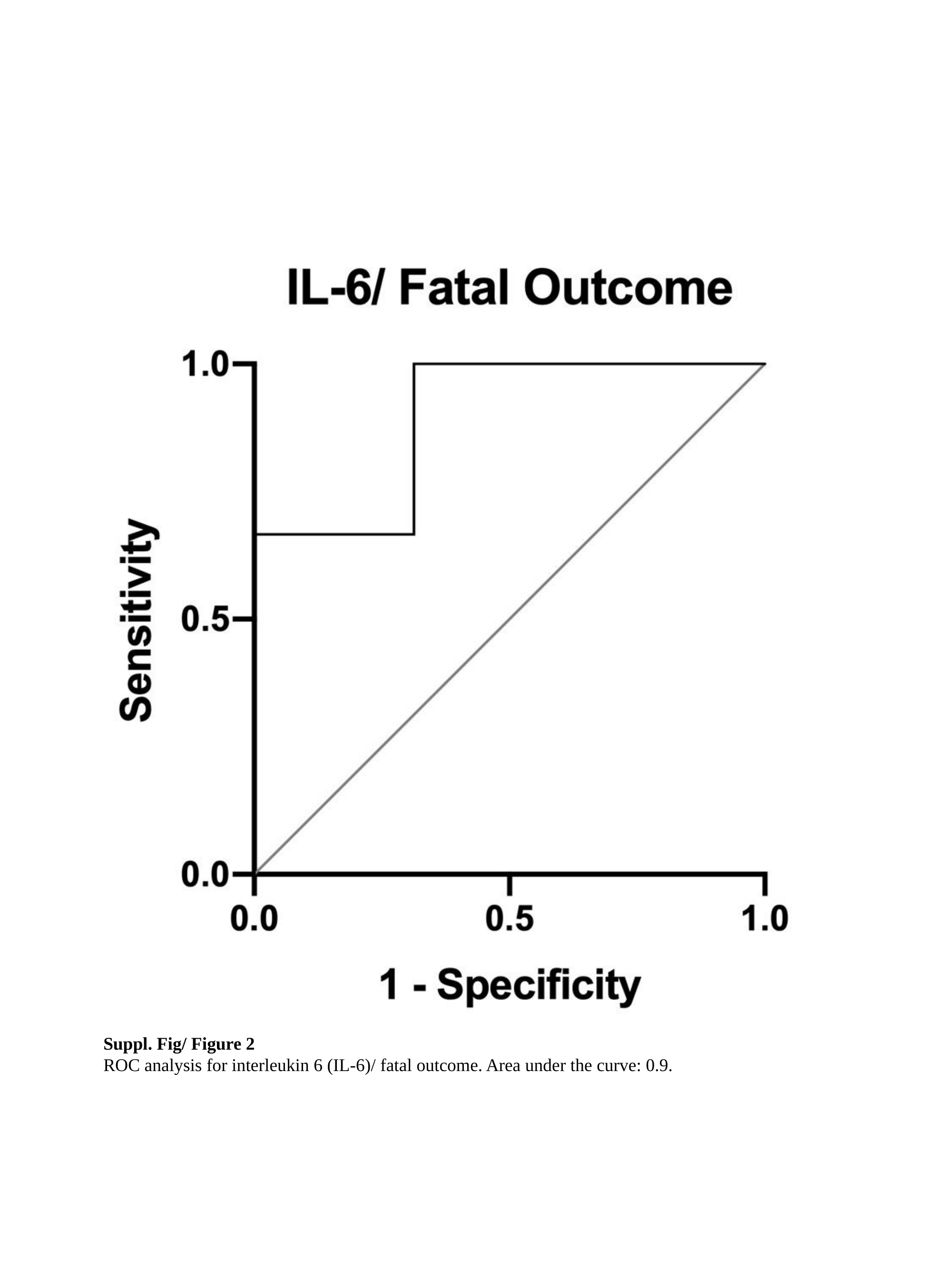

Suppl. Fig/ Figure 2
ROC analysis for interleukin 6 (IL-6)/ fatal outcome. Area under the curve: 0.9.
