# Supplementary material for: The Prognostic Value of Troponin in Pediatric Polytrauma
Source: Front Pediatr. 2019 Nov 20;7:477. doi: 10.3389/fped.2019.00477 (PMC6879657; doi:10.3389/fped.2019.00477)
Supplement: Supplementary file 2 [file Table_1.DOCX]

**Suppl. Tables/ Table 1**

Correlation analyses of interleukin 6 (IL-6) levels in patients’ plasma with creatine-kinase activity levels (CK-activity), lactate levels, Injury Severity Score (ISS) values, length of stay on the intensive care unit (ICU) and stratification of cases into two groups (IL-6 positive and negative) with comparison for parameters of interest. ^1^: depicts the number of cases for which data was available; ^2^: due to low case numbers, data should be interpreted with caution; n.s.: not significant.

|  | **n (included cases)^1^** | **Spearman's r** | **p-value** |  |
| --- | --- | --- | --- | --- |
| **ISS** | 35 | 0.234 | n.s. |  |
| **CK** | 35 | 0.728 | p<0.001 |  |
| **Lactate** | 13^2^ | - 0.095 | n.s. |  |
| **Length of ICU** | 34 | 0.006 | n.s. |  |
|  |  |  |  |  |
|  |  |  |  |  |
| **Analysis of IL-6 groups (cut-off: 7 pg/mL)** | | |  |  |
|  |  |  |  |  |
| **Parameter** | **IL-6 negative** | **IL-6 positive** | **p (Mann-Whitney U test)** | |
| **IL-6 (median (1./ 3. Qrt); pg/ mL)** | 2 (0/ 4) | 76 (30/ 162) | p<0.001 |  |
| **ISS (median (1./ 3. Qrt))** | 26 (10/ 29) | 18 (15/ 28) | 0.815 |  |
| **TnT (median (1./ 3. Qrt); ng/ mL)** | 4 (4/ 5) | 8 (1/ 23) | 0.385 |  |
| **CK (median (1./ 3. Qrt); U/ L)** | 242 (161/ 415) | 459 (237/ 1360) | 0.122 |  |
| **Lactate (median (1./ 3. Qrt); mmol/ L)** | 1.8 (1.6/ n/a) | 1.4 (0.9/ 2) | 0.161 |  |
| **Length of ICU (median (1./ 3. Qrt); days)** | 4 (3/ 8) | 2 (1/ 5) | 0.364 |  |

**Suppl. Tables/ Table 2**

Association analysis for troponin T (TnT) plasma levels elevated above the cut-off of 14 ng/ mL. ^1^: statistical association analysis with Chi-Square test; ^2^: depicts the number of cases for which data was available; n.s.: not significant.

|  | **Thorax Trauma** | |  |
| --- | --- | --- | --- |
|  | not reported | reported |  |
| **n (included cases)^2^** | 11 | 21 |  |
| **Above cut-off/ Cases (fraction)** | 2/11 (0.18) | 10/21 (0.47) | n.s.^1^ |
|  |  |  |  |
|  | **Lung Contusion** | |  |
|  | not reported | reported |  |
| **n (included cases)^2^** | 18 | 14 |  |
| **Above cut-off/ Cases (fraction)** | 3/18 (0.17) | 7/14 (0.5) | p=0.044^1^ |
|  |  |  |  |
|  | **SOFA Score** | |  |
|  | 0-2 | >2 |  |
| **n (included cases)^2^** | 21 | 11 |  |
| **Above cut-off/ Cases (fraction)** | 4/21 (0.19) | 7/11 (0.64) | p=0.012^1^ |
|  |  |  |  |
|  | **Fatal Outcome** | |  |
|  | survivor | non-survivor |  |
| **n (included cases)^2^** | 31 | 3 |  |
| **Above cut-off/ Cases (fraction)** | 9/31 (0.29) | 3/3 (1.0) | p=0.014^1^ |
